# Evaluation of stroke pre-hospital management in Lebanon from symptoms onset to hospital arrival and impact on patients’ status at discharge: a pilot study

**DOI:** 10.1186/s12883-022-03018-0

**Published:** 2022-12-20

**Authors:** Hiba Kamal, Sara Assaf, Mayssan Kabalan, Raneem El Maissi, Dima Salhab, Deema Rahme, Nathalie Lahoud

**Affiliations:** 1 Faculty of Public Health, Lebanese University, Fanar, Lebanon; 2 Faculty of Pharmacy, Lebanese University, Hadath, Lebanon; 3 Pharmacy Practice Department, Faculty of Pharmacy, Beirut Arab University, Beirut, Lebanon

**Keywords:** Stroke, Cerebrovascular accident (CVA), Emergency medical services, Tissue plasminogen activator, Lebanon

## Abstract

**Background:**

Hospital arrival time after acute ischemic stroke onset is the major factor limiting the eligibility of patients to receive intravenous thrombolysis. Shortening the prehospital delay is crucial to reducing morbidity and mortality for stroke patients. The study was conducted to investigate the factors that influence hospital arrival time after acute stroke onset in the Lebanese population and to assess the effect of the prehospital phase on patients’ prognosis at discharge.

**Method:**

A prospective cross-sectional study was performed in eleven hospitals from April to July 2021 including 100 patients having stroke symptoms or transient ischemic attack (TIA). Two questionnaires were used to collect data addressing patient management in the pre-hospital phase and the in-hospital phase. Descriptive and bivariate analyses were done to evaluate the potential associations between prognosis, pre-hospital characteristics, and other factors.

**Results:**

The patients’ mean age was 70.36 ± 12.25 years, 43 (53.8%) of them were females, and 79 (85%) arrived within 3 hours after symptoms onset. Diabetic patients had a significant delay in hospital arrival compared with non-diabetics (27.0%vs.7.1%, *p*-value = 0.009). Moreover, 37 (75.5%) of school-level education patients arrived early at the hospital compared to 7 (100%) of university-level education (*p*-value = 0.009). The modified Rankin Scale (mRS) at discharge in patients with hemorrhagic stroke (10 (90%)) was worse than that in patients with ischemic stroke (38 (80%)) or TIA (3 (15%)) (*p*-value< 0.001).

**Conclusion:**

The study findings make it imperative to raise awareness about stroke symptoms among the Lebanese population. Emergency Medical Services should be utilized appropriately in the transportation of stroke patients to achieve optimal patient outcomes.

## Impact of the findings on clinical practice

- The management of acute stroke is a time-dependent process where the time between symptoms onset and hospital arrival is crucial to determine the eligibility for fibrinolytic administration and hence reducing the resulting disability for ischemic stroke patients.
- This is the first study in Lebanon to assess the pre-hospital phase management of stroke patients.
- Nationwide awareness campaign is needed to improve the public knowledge of stroke and the importance of timely treatment when acute stroke symptoms are suspected.
- Strategies to improve stroke awareness and referral protocols together with the use of telemedicine and mobile stroke units should be implemented to reduce the hospital arrival time

## Introduction

Stroke is a serious medical emergency that contributes to significant morbidity and mortality. It is the second leading cause of death and the third leading cause of disability globally [1, 2]. Lebanon has an age and sex-adjusted stroke prevalence of 0.5%. This prevalence increases with age for both males and females and differs between governates with probable high stroke mortality in suburbs [3]. Hypertension (75.2%), low levels of physical activity (89.4%), and diabetes mellitus (40.9%) are the most prevalent risk factors among Lebanese patients [4]. Concerning the treatment of acute ischemic stroke, intravenous administration of recombinant tissue plasminogen activator (rt-PA), alteplase, is considered the leading treatment for acute stroke management. It has been shown in randomized controlled trials to decrease functional disability with an absolute risk reduction of 7–13% relative to placebo when it is given within 4.5 hours after the onset of ischemic stroke [5–8]. The efficacy of alteplase decreases rapidly with time elapsed from symptom onset and this increases the risk of complications for the patients [9]. Although efforts have been made to reduce delays in hospital admission in patients with stroke, at least half of patients with major ischemic stroke arrive later than 4.5 hours after stroke onset and are therefore not eligible for fibrinolysis [10]. For example, in Thailand, only 3.8 to 5.5% of ischemic stroke patients received intravenous rt-PA [11, 12]. This delay in the arrival of acute stroke cases may be attributed to several factors including awareness of stroke signs and symptoms, perception of the importance of early presentation for treatment, religious and cultural beliefs, educational level, geographical accessibility, and technical factors such as the availability of diagnostic facilities and therapies [13–17]. This raises the need to exert more efforts to minimize the onset-to-door time (ODT); which is the time between the stroke onset and the time the patient is admitted to the hospital, to initiate treatment as early as possible thus achieving better patient prognosis. A study done in Spain showed that an arrival time of less than 3 hours was associated with lower mortality at 3 and 6 months and with fewer in-hospital complications [18]. According to a study aimed to assess the obstacles to acute ischemic stroke management in Lebanon, only four patients (2.5%) received fibrinolytic therapy. Furthermore, more than 55% of the stroke patients were dependent at discharge, as represented by mRS score of 0–2, whereas 33% of them were independent (mRS score of 3–5) [19].

However, the factors affecting the management of stroke in the Lebanese population were not addressed so far, therefore, this study aimed to investigate the factors that significantly influence hospital arrival time after acute stroke in the Lebanese population and to shed light on the effect of this prehospital phase on patients’ prognosis at discharge.

## Methods

A prospective cross-sectional study was performed in eleven hospitals to collect data for patients presenting with stroke symptoms or TIA during the period from April to July 2021. The included hospitals were divided between private (*n* = 9) and public (*n* = 2). Eight of them were located in Bekaa while the rest were in Beirut. The study was approved by the Institutional Review Board (IRB) of The Psychiatric Cross Hospital and all methods were performed in accordance with the relevant guidelines and regulations.

### Participants

Twenty-four hospitals specifically in Bekaa and Beirut (which made approximately the total number of hospitals in these two regions) were chosen for patient recruitment due to the transportation limitation that made access to hospitals in other regions difficult for our team during the period of data collection. However, only 11 hospitals were included, The 13 remaining hospitals were not included for different reasons such as hospital refusal to participate (*n* = 6), long approval procedures (*n* = 2), not admitting stroke patients at the time of approach (*n* = 2), geographical limits (*n* = 2), and lack of cooperation (*n* = 1).

One hundred patients were randomly selected from which ninety-three were included. The inclusion criteria for patients were those 18 years old and above and presenting with at least one of the symptoms related to stroke such as; sudden weakness or numbness of the face, arm, or leg, most commonly on one side of the body, trouble speaking or understanding speech or other symptoms that may include: confusion, difficulty in speaking, or understanding speech [2]. Their admission was confirmed by the physician in charge with suspicion of stroke, or following a confirmation based on performing a brain CT or magnetic resonance imaging (MRI). Patients who were not diagnosed with stroke were not followed during their hospital stay and discharge.

1. **Data Collection**

An average of 3 visits per week were done in every selected hospital, targeting Critical Care Units (intensive care units-cardiac care units) and the medical departments. Clinical information at arrival was retrieved from the medical charts or through direct interviews with the patients or their family members. The collected indicators were the patient’s symptoms on arrival at the hospital according to FAST (face, arm, speech impairment, time to call), the final diagnosis, and the type of stroke (confirmed by imaging). The patient’s past medical history such as stroke risk factors, lifestyle, and the mRS assessment for neurologic disability was also included in the study. Stroke risk factors included diabetes, hypertension, atrial fibrillation, hyperlipidemia, kidney failure, heart failure, and a family history of stroke. These factors were assessed by asking the patients or their family members if they were diagnosed by a specialist with any of the mentioned health conditions. A current smoker label is assigned if a patient was a smoker within the past year while a quitter smoker label is assigned if a patient has not smoked for at least 1 year. To assess the in-hospital management, the following parameters were collected: the door-to-imaging time (DIT), the door-to-treatment time (DTT) in minutes, and the mRS at discharge. In unconscious patients, consent and data were collected from their caregivers and those who transported the patients to the hospital to get the most accurate answers.

2) **Variables included in the questionnaire**

The first action at the stroke onset (calling emergency, a driver, or starting transportation of the patients to the hospital), the mean of transportation, the availability of a family member at the onset, the time interval between onset to call (OCT), the onset to door time (ODT), and other factors that might affect the stroke management were gathered to assess the prehospital phase. Moreover, medical charts, consultations, and direct interviews aimed to collect patients’ clinical information and medical history, the door-to-imaging time (DIT), and the door-to-treatment time (DTT) by minutes.

3) **Data sources**

The mRS at arrival and discharge was measured by a trained investigator according to the state of the patient. This scale allows clinicians to measure the degree of dependence or disability in the daily activities of people who have suffered a stroke or other causes of neurological disability [16]. It is a 7-level ordered scale capturing levels of patient functional dependence with scores ranging from 0 (fully independent) to 6 (dead). In addition patients and their families were asked for additional information about their ability to spot stroke signs and when to call for emergency support (F.A.S.T) [17].

### Statistical analysis

Data were analyzed using the IBM Statistical Package for Social Sciences (SPSS), version 21.0.0. Means and standard deviations were used to describe continuous variables for the descriptive analysis, while frequencies and percentages were used to represent categorical ones. To explore potential associations between prognosis, pre-hospital parameters, and other factors, two main dependent variables were considered:

The ODT is dichotomized to ≤3 hours (early arrival) and > 3 hours (late arrival)); this cut-point is justified by the fact that intravenous injection of rt-PA is effective in patients with all types of acute ischemic stroke when administered within 4.5 h of symptoms onset [8]. Besides, the mRS at discharge transformed from a scale (0 to 6) to a binary variable with a score of 2 as the cutoff point (no or slight symptoms and moderate or severe symptoms to death). Some further variables were dichotomized such as DIT (≤20 min, > 20 min) and DTT (≤60 min, > 60 min) and this is according to the American Stroke Association (ASA) 2018 guidelines. OCT was dichotomized by its 10 minutes median. We conducted a bivariate analysis using the Student T-test to compare means (quantitative variables) and the Pearson Chi-square test (or exact Fisher’s test as appropriate) to compare percentages (categorical variables). Post-hoc test with Bonferroni correction was performed for all multinomial variables having significant *p*-values, where a *p*-value less than 0.05 was considered statistically significant.

## Results

Patients’ mean age was 70.36 ± 12.25 years, including 43 (53.8%) females, and the majority (89.2%) were Lebanese. Demographic characteristics of the patients were presented in Table 1 with a comparison between groups for early and delayed arrival based on ODT results.

**Table 1:** Patients’ Demographic Characteristics With a Comparison between Groups for Early and Delayed Arrival to Hospital

Demographic Characteristics		ODT		***p***-value
	N (%)	Early 79/93 (85%)	Late 14/93 (15%)	
Age (years) Mean ± standard deviation	70.36 ± 12.25	70.13 ± 12.54	69.64 ± 11.00	.893
Gender				
Male	50/93 (53.8%)	40/50 (80%)	10/50 (20%)	.150
Female	43/93 (46.2%)	39/43 (90.7%)	4/43 (9.3%)	
Nationality				
Lebanese	83/93 (89.2%)	71/83 (85.5%)	12/83 (14.5%)	.643
Others	10/93 (10.8%)	8/10 (80%)	2/10 (20%)	
Housing situation				.448
Alone	11/93 (11.8%)	11/11 (100%)	0/11 (0%)	
With family	82/93 (88.2%)	68/82 (83%)	14/82 (17%)	
Patient Education				.029
Illiterate	37/93 (39.8%)	35/37 (94.6%)	2/37 (5.4%)	.035
School	49/93 (52.7%)	37/49 (75.5%)	12/49 (24.5%)	.009†
University	7/93 (7.5%)	7/7 (100%)	0/7 (0%)	.271
Attendant education				
Illiterate	17/93 (18.3%)	15/17 (88.2%)	2/17 (11.8%)	.769
School	56/93 (60.2%)	46/56 (82.1%)	10/56 (17.9%)	
University	20/93 (21.5%)	18/20 (90%)	2/20 (10%)	
Employment status				
Employed	23/93 (24.7%)	20/23 (87%)	3/23 (13%)	.442
Unemployed^a^	70/93 (75.3%)	59/70 (84.3%)	11/70(15.7%)	
Monthly income				
< 750,000 L.L.	54/93 (58.1%)	43/54 (79.6%)	11/54 (20.4%)	.352
750,000-2,250,000 L.L.	35/93 (37.6%)	32/35 (91.4%)	3/35 (8.6%)	
> 2,250,000 L.L.	4/93 (4.3%)	4/4 (100%)	0/4 (0%)	
Payer				
Governmental insurance^b^	80/93 (86%)	68/80 (85%)	12/80 (15%)	.809
Non-Governmental insurance (UN^c^)	9/93 (9.7%)	7/9 (77.8%)	2/9 (22.2%)	
Private insurance	2/93 (2.2%)	2/2 (100%)	0/2 (0%)	
Out-of-Pocket (No insurance)	2/93 (2.2%)	2/2 (100%)	0/2 (0%)	

ODT (onset to door time)

^a^includes Housewife/Retired/Unemployed

^b^includes NSSF (National Social Security Fund)/COOP (The Cooperative of the Civil Servants) /Military/MOPH (Ministry of Public Health) /ISF (Internal Security Forces)

^c^UN (United Nations)

†post-hoc analysis

There were several factors related to the delayed ODT. Among the medical reasons, patients with diabetes mellitus were more likely to have a delayed hospital arrival than non-diabetic patients (27.0% vs. 7.1% respectively, *p*-value = 0.009), whereas other risk factors including a history of a previous stroke were not significantly associated with ODT. Concerning stroke types, 43(46.2%) patients had an ischemic stroke, 18(19.3%) had a transient ischemic attack, 11 (11.8%) had an acute hemorrhagic stroke, and 21 (22.5%) had stroke-like symptoms but were not diagnosed with any type of stroke (as illustrated in Table 2). As for the non-medical factors related to ODT, results showed that patients who tended to take the first action towards transport were more likely to arrive earlier at the emergency department (ED) than those who didn’t (92.3% vs 66.7%, *p*-value = 0.004). However, the means of transportation to the hospital was not significantly associated with ODT. The non-medical factors related to ODT with a comparison between groups for early and delayed hospital arrival were demonstrated in Table 3.

**Table 2:** Stroke Risk Factors among Patients with a Comparison between Groups for Early and Delayed Arrival

Stroke Risk Factors		ODT		***p***-value
	N (%)	Early 79/93 (85%)	Late 14/93 (15%)	
Diabetes				0.009
Yes	37/93 (39.8%)	27/37 (73.0%)	10/37 (27.0%)	
No	56/93 (60.2%)	52/56 (92.9%)	4/56 (7.1%)	
Hypertension				0.536
Yes	63/93 (67.7%)	52/63 (82.5%)	11/63 (17.5%)	
No	30/93 (32.3%)	27/30 (90.0%)	3/30 (10.0%)	
Atrial fibrillation				1.000
Yes	5/93 (5.4%)	5/5 (100.0%)	0/5 (0.0%)	
No	88/93 (94.6%)	74/88 (84.1%)	14/88 (15.9%)	
Hyperlipidemia				0.716
Yes	17/93 (18.3%)	14/17 (82.4%)	3/17 (17.6%)	
No	76/93 (81.7%)	65/76 (85.5%)	11/76 (14.5%)	
Kidney failure				1.000
Yes	2/93 (2.2%)	2/2 (100.0%)	0/2 (0.0%)	
No	91/93 (97.8%)	77/91 (84.6%)	14/91 (15.4%)	
Smoking				0.609
Yes	71/93 (76.3%)	63/71(88.7%)	8/71 (11.3%)	
No	22/93 (23.7%)	16/22 (72.7%)	6/22 (27.3%)	
Heart Failure				1.000
Yes	7/93 (7.5%)	7/7 (100%)	0/7 (0%)	
No	86/93 (92.5%)	72/86 (83.7%)	14/86 (16.3%)	
Family history of stroke				0.608
Yes	11/93 (11.8%)	8/11 (72.7%)	3/11 (27.3%)	
No	82/93 (88.2%)	71/82 (86.6%)	11/82 (13.4%)	
Disabling Symptoms				
Yes	48/93 (51.6%)	43/48 (89.6%)	5/48 (10.4%)	0.197
No	45/93 (48.3%)	36/45 (80%)	9/45 (20%)	
Previous Stroke				
Yes	25/93 (26.8%)	19/25 (76%)	6/25 (24%)	0.190
No	68/93 (73.1%)	60/68 (88.2%)	8/68 (11.7%)	
mRS at arrival				
No or slight symptoms	20/93 (21.5%)	17/20 (85%)	3/20 (15%)	1.000
Moderate to severe	73/93 (78.5%)	62/73 (84.9%)	11/73 (15.1%)	
Final Diagnosis				
Not stroke	21/93 (22.5%)	19/21 (90.5%)	2/21 (9.5%)	
TIA	18/93(19.3%)	16/18 (88.9%)	2/18 (11.1%)	0.764
Acute Hemorrhagic stroke	11/93 (11.8%)	9/11 (81.8%)	2/11 (18.2%)	
Ischemic stroke	43/93 (46.2%)	35/43 (81.4%)	8/43 (18.6%)	

ODT Onset to door time

**Table 3:** Non- Medical Factors Related to Onset to Door Time among Patients with a Comparison between Groups for Early and Delayed Arrival

		ODT		***p***-value
Factors	N (%)	Early 79/93(85%)	Late 14/93 (15%)	
1st action taken was toward transport				
Yes	66/93 (70.9%)	61/66 (92.4%)	5/66 (7.5%)	0.004
No	27/93 (29.1%)	18/27 (66.7%)	9/27 (33.3%)	
Way of transportation				
Ambulance	29/93 (31.2%)	26/29 (89.7%)	3/29 (10.3%)	0.537
Private	64/93 (68.8%)	53/64 (82.8%)	11/64 (17.2%)	
Bystander’s awareness				
Yes	44/93 (47.3%)	36/44 (81.8%)	8/44 (18.2%)	0.135
No	49/93 (52.7%)	43/49 (87.7%)	6/49 (12.3%)	
Patient Info (F.A.S.T)				
Yes	26/93 (28%)	19/26 (73.1%)	7/26 (26.9%)	0.083
No	67/93 (72%)	60/67 (89.6%)	7/67 (10.4%)	

*ODT* Onset to door time, *mRS* Modified Rankin Scale, *TIA* Transient ischemic attack

Pre-hospital and in-hospital managementfactors were highly associated with stroke outcome which was presented by mRS at discharge. The delay in onset-to-call time, door-to-imaging time, and door-to-treatment time resulted in significantly worse mRS at discharge. Moreover, patients with acute hemorrhagic stroke had the worst outcome compared to patients having other stroke types. The characteristics of pre-hospital and in-hospital early management were presented in Table 4.

**Table 4:** Characteristics of Pre and In-Hospital Early Management and Association with Modified Rankin Scale at Discharge among Stroke Patients

Variables		mRS at discharge		***p***-value
	N (%)	No to low symptoms 28/79 (35.4%)	Moderate or severe to death 51/79 (64.6%)	
DTT				
> 60 min	47/79 (59.5%)	9/47 (19.1%)	38/47 (80.9%)	0.015
≤ 60 min	32/79 (40.5%)	19/32 (59.4%)	13/32 (40.6%)	
OCT				
> 10 min	34/79 (43%)	3/34 (8.8%)	31/34 (91.2%)	0.024
≤ 10 min	45/79 (57%)	25/45 (55.6%)	20/45 (44.4%)	
ODT				
> 3 h	14/79 (17.7%)	2/14 (14.3%)	12/14 (85.7%)	0.186
≤ 3 h	65/79 (82.3%)	39/65 (60%)	26/65 (40%)	
DIT				
> 20 min	53/79 (67.1%)	12/53 (22.6%)	41/53 (77.3%)	0.008
≤ 20 min	26/79 (32.9%)	16/26 (61.5%)	10/26 (38.5%)	
Comorbidities				
Yes	61/79 (77.2%)	18/61 (29.5%)	43/61 (70.5%)	0.120
No	18/79 (22.8%)	10/18 (55.6%)	8/18 (44.4%)	
Stroke Subtypes				
TIA	20/79 (25.3%)	17/20 (85%)	3/20 (15%)	< 0.001†
Hemorrhagic stroke	11/79 (13.9%)	1/11 (9.1%)	10/11 (90.9%)	< 0.001
Ischemic stroke	48/79 (60.8%)	10/48 (20.8%)	38/48 (79.2%)	.089 < 0.001

*mRS* Modified Rankin Scale, *DTT* Door to treatment time, *OCT* Onset to call time, *ODT* Onset to door time, *DIT* Door to imaging time, *TIA* Transient ischemic attack

†Post hoc analysis

## Discussion

The results of the study revealed that the majority of the stroke patients arrived within 3 hours after the onset of symptoms and were transported by their families or caregivers. Patients with a school-level education arrived early at the ED while patients with diabetes mellitus were more likely to have a delayed arrival. Patients who tended to take the first action towards transport were more likely to arrive earlier at ED. Results also showed that patients who took more than 10 minutes to call for transport, who underwent late cerebral imaging, and who received late treatment had significantly worst mRS at discharge. Concerning the subtypes of stroke, patients with hemorrhagic stroke had worse mRS at discharge than patients with ischemic stroke or TIA.

These results are consistent with the results of two studies done in Turkey showing that 72.6% [20] and 68.4% [21] of patients arrived within 3 hours from symptoms onset. However, results from other countries reporting the percentage of patients arriving within 3 hours from symptoms onset were as follows: 23% in France [18], 25% in China [22], 28% in Italy [23], 29% in India [24], 32.5% in Korea [25], 40.2% in Thailand [26], and 46% in the USA [27]. Moreover, 29 (30%) stroke cases were transported to the hospital by ambulance while the majority (64 (70%)) were transported by their families and/or caregivers). This is consistent with a study done in Lebanon that showed that only 23% of the cases were transported by ambulance [28]. These results can reflect a low level of awareness on how to manage emergencies among the Lebanese population.

Our study showed that the percentage of patients with school-level education that arrived early atthe ED was significantly lower than those of illiterate and university educational levels. However, Odiase et al. And Wahab et al. Proved that a higher level of education is a predictor of stroke knowledge [29, 30]. This may be due to the low sample size in the current study and the insufficient number of university graduates in our sample (7 patients), which may mask the effect of higher educational levels on early arrival. It is important to note that all patients (100%) having a university educational level were admitted early to hospitals, but this result was not significant (*p*-value = 0.271). According to the fact that early transport shortens the ODT, patients who tended to take the first action towards transport were more likely to arrive at the ED faster than those who didn’t (*p*-value = 0.004). This came in agreement with a study done by Puolakka et al. Where OCT was the single most important determinant of early hospital arrival and treatment [29].

On the other side, patients with diabetes mellitus were more likely to have a delayed arrival (*P* = 0.009). One potential explanation would be that patients who have diabetes might present symptoms congruent with those of stroke, thus they or their caregivers might have interfered based on hypoglycemia and not stroke and this can lead to a misperception and neglection of the seriousness of the condition instead of immediately moving to the ED. This result is consistent with a study done by Homoud et al. Where a significant majority of the non-diabetic stroke patients presented to the ED within 3 hours of symptom onset (*P* = 0.003), whereas almost 38% of diabetic stroke patients presented after 24 hours of symptom onset [31].

Furthermore, the study highlighted the critical impact of pre-hospital management on the patient’s prognosis upon discharge. A significant association was found between the delay in calling for patient transport (OCT) and the worst mRS upon discharge (*p*-value 0.015). Cutting the time to call for a transport action after the onset of symptoms is logically expected to cut the ODT which, in turn, represents the total duration of the pre-hospital management phase. Our study also showed that patients who were late to take action towards treatment, be it the call action or the door to treatment time, did have poorer mRS at discharge. This association was seen in patients who underwent late cerebral imaging (*p*-value = 0.008), and those who received late treatment (*p*-value = 0.015). This came in agreement with two studies that stressed the effect of ODT and DTT [32, 33]. Moreover, another study showed that swift action toward initiating the treatment protocol at hospitals has a positive impact on the case prognosis [34]. In addition, The prehospital phase of the stroke chain of survival also showed that the OCT is an integral component of the ODT and thus shortening the OCT is expected to improve the case prognosis upon discharge [35]. Our results also came in agreement with the relationship between prehospital notification and improving stroke outcomes study and it showed that patients with a shorter ODT left the hospital with a good outcome [36]. This highlights the importance of taking immediate action to call for transport due to its proven effect on the ODT and therefore on the patient’s quality of life [37]. Regarding the subtypes of stroke, our results were in line with a Canadian study’s findings that patients presenting with a hemorrhagic stroke had the worst outcomes upon discharge [38].

The present study had some limitations. Firstly, the sample size was low due to the time limit, the low incidence of stroke cases at the time of the study, and the refusal of some hospitals to cooperate, and thus several university hospitals were not included. Secondly, hospital recruitment was not done randomly. Therefore, our results cannot be generalized to the general patient population in the country since we couldn’t include enough university hospitals or hospitals equipped with stroke centers. Another limitation was the inaccurate reporting of treatment and interventions by the medical staff leading to inaccurate door-to-treatment intervals. Furthermore, the study did not follow up on the case prognosis after exiting the hospitals but was rather limited to the mRS at discharge.

Despite these limitations, this is the first study done in Lebanon discussing the pre-hospital phase management of stroke patients. In addition, we included 11 different hospitals and adopted a prospective follow-up approach to the patients during their stay which provided an entry gate into studying the pre-hospital management of acute stroke cases in Lebanon. Thus, it paves the way for future studies on a national cohort of patients to confirm our findings. Furthermore, patients admitted with a suspicion of stroke or transient ischemic attack (TIA), and those who underwent brain CT scan or MRI to rule-out stroke were included in the study, which added more value to the concept of “pre-hospital management” of cases presenting to the ER.

## Conclusion

The preliminary results of this pilot study showed the clear need to raise awareness about stroke symptoms and the need to take immediate action toward case transport. Patients especially those with comorbidities along with their caregivers have to be concerned with health promotion or educational programs to decrease the time of transport and arrival to the ED. Concerning hospitals, effective in-hospital management is required to ensure the prompt diagnosis and treatment of patients with acute ischemic stroke and thus improve stroke patients’ prognosis. Larger studies are needed to support these results, highlighting the need for pre-hospital management improvement in the Lebanese population.

## Data Availability

All data generated and analyzed during this study are included in this published article. Any further data can be provided by the corresponding author upon request.
